# Short- and long-term reproducibility of peripheral superficial vein depth and diameter measurements using ultrasound imaging

**DOI:** 10.1186/s12880-022-00945-9

**Published:** 2022-12-02

**Authors:** Matsumoto Miharu, Hashiguchi Nobuko, Kobayashi Hiromitsu

**Affiliations:** 1 Department of Health Sciences, Kyushu University, Japan 3-1-1, Maidashi, Higashi-Ku, Fukuoka City, 812-8582 Japan; 2 Ishikawa Prefectural Nursing University, Japan 1-1 Gakuendai, Kahoku, Ishikawa 929-1210 Japan

**Keywords:** Ultrasound imaging, Intraclass correlation coefficients, Coefficient of variation

## Abstract

**Background:**

Ultrasound imaging is used for diagnosis, treatment, and blood vessel visualization during venous catheter placement. However, various physiological factors (e.g., body temperature and exercise) influence vein diameters, which are expected to exhibit daily or diurnal fluctuations. Therefore, this study aimed to determine the intraday (short-term) and interday (long-term) reproducibility of repeated measurements of the depth and diameter of peripheral superficial veins.

**Methods:**

Twenty-three healthy young women (mean age, 21.7 ± 0.8 years) participated in the study to examine the short- and long-term reproducibility of the depth and diameter of the cutaneous vein in the left elbow fossa acquired by ultrasound imaging. Short-term measurement intervals were 10 s, and the probe was released from the skin for each acquisition, which was repeated five consecutive times. Long-term measurements were performed at the same time on the next day following the same procedure. The acquired images were analyzed for vein depth and diameter using ImageJ software. The intraclass correlation coefficient (ICC) was calculated to determine the short- and long-term reproducibility of the measurements. The relationship between the venous depth and venous diameter intra-individual variation was analyzed, as well as the influence of body composition (body fat and muscle mass) on the venous diameter and depth.

**Results:**

For vein depth measurements, the short- and long-term ICCs were 0.94–0.96 and 0.88, respectively. For the vein diameter, the short- and long-term ICCs were 0.94–0.97 and 0.67, respectively. The short-term ICCs for both vein depth and diameter exceeded 0.9, indicating that the ultrasound vascular measurement was sufficiently reliable. However, long-term reproducibility was slightly lower, especially for the vein diameter. No correlation was found between the intra-individual variation of the vein diameter and vein depth. Although the vein diameter and body fat mass uncorrelated, the vein depth and body fat mass significantly correlated (r = 0.675, 95% confidence interval = 0.281–0.830).

**Conclusions:**

The long-term reproducibility of vein diameters was somewhat lower than that of the short-term reproducibility. This could be attributed to fluctuations in the physiological state of the participant rather than to the instability of the measurement. Therefore, ultrasound measurement of the peripheral superficial vein is sufficiently reliable.

## Background

Ultrasound imaging is an essential technique for diagnosis and treatment. Unlike computed tomography, X-ray, or magnetic resonance imaging, ultrasound imaging does not require large equipment and is free from the risk of X-ray exposure. Therefore, ultrasound imaging at the bedside is relatively easy, and a timely diagnosis can be made owing to real-time image acquisitions. Specifically, ultrasound imaging technology has evolved significantly over the past two decades, with devices becoming smaller and lighter [1], enabling its applications in various healthcare fields [2]. For example, it can be used for the assessment of cardiac dynamics in patients with heart failure and dialysis, focused assessment with sonography for trauma in the emergency room, and examination of bladder capacity and constipation [3–6]. In these imaging procedures, inter- and intra-observer agreements are important because the measurement accuracy might depend on the observer [7, 8].

Ultrasound imaging can be also utilized for blood vessel visualization during venous catheter placement [9]. Ultrasound-guided catheter insertion into difficult-to-access veins improves success rates compared with conventional blind insertion [10, 11]. This method can be also used to evaluate venous vasodilation interventions. Vessels with higher success rates for peripheral vein catheter insertion are more visible and palpable [9, 12, 13] and have larger diameters [14, 15]. Therefore, in previous studies on various interventions to facilitate venipuncture (e.g., local heating and postural changes), the vein diameter was used to evaluate their effectiveness [16–20].

Vein diameters are known to exhibit diurnal fluctuations [21]. Specifically, the inferior vena cava and peripheral veins of the lower extremities show great physiological variabilities [22, 23].

Several studies have been made on the intra- and inter-observer agreement of ultrasound vascular measurements, and the results were nearly perfect for intra-observer and considerably high for inter-observer [24–27]. Therefore, the accuracy of vein measurement depends on the magnitude of vasomotion, not on the reliability of the observer.

This study aimed to determine the intraday (short-term) and interday (long-term) reproducibility of repeated measurements of the depth and diameter of peripheral superficial veins.

## Methods

### Participants

The study enrolled 23 healthy young women who were not patients, smokers, or pregnant. Table 1 presents the demographic characteristics of the participant. The sample did not include obese individuals. During the study period, alcohol consumption was prohibited, and the participants were required to sleep at least 6 h beforehand. The participants also avoided strenuous exercise after waking up on the experiment day, finished eating at least 2 h before the experiment, and limited intake of stimulants and caffeine.

**Table 1:** Demographic characteristics of the participants (n = 23)

	Mean ± SD
Age	21.7 ± 0.8
Height (cm)	158.3 ± 5.7
Weight (kg)	50.9 ± 4.5
Body mass index	20.4 ± 1.8
Fat mass (kg)	13.8 ± 2.9
Muscle mass (kg)	33.9 ± 2.5

*SD* standard deviation

The study was approved by the ethics committee of the university where the study was conducted. The researchers explained the study’s content and obtained verbal and written informed consent before starting the study.

### Procedures

This study was conducted for two consecutive days in October 2021. The air temperature in the laboratory was maintained at 25 °C. Initially, the participants changed into T-shirts and shorts made of the same material, and their body temperature, pulse rate, blood pressure, and height were measured. In addition, the total body muscle mass and body fat were recorded using a body composition analyzer (Inbody 270; Inbody, Tokyo, Japan). Then, the participants sat on a chair and placed their forearms on an 83-cm-high platform for 15 min. The elbow joint was aligned with the mark on the table, the forearm was kept extended, and the arm was fixed with a belt to prevent the arm from moving.

The same operator obtained B-mode transverse images of the median cutaneous vein or radial vein of the left elbow fossa by ultrasound imaging (Versana Active; GE Health Care, Tokyo, Japan) with a 10–12 MHz linear probe. During measurements, the probe was fixed to the stand and maintained at the same height. A 1-cm thick layer of gel was used to avoid compressing the vein with the probe’s pressure. The same probe was used for all measurements, and the scanner was set to an observable image depth of 20 mm. Gain was optimized for each participant. The skin at the probe application site was marked, and the beam was applied to the same vein each time. The interval between each imaging acquisition was approximately 10 s, and the probe was released once for each acquisition.

### Image analysis

All images were saved on the device and converted to jpeg files, and these jpeg files were converted to 256-bit grayscale using ImageJ [28], a public domain software used for processing and analyzing scientific images: 1 pixel = 0.034 mm The images were analyzed at a resolution of 0.034 mm.

**Fig. 1:**
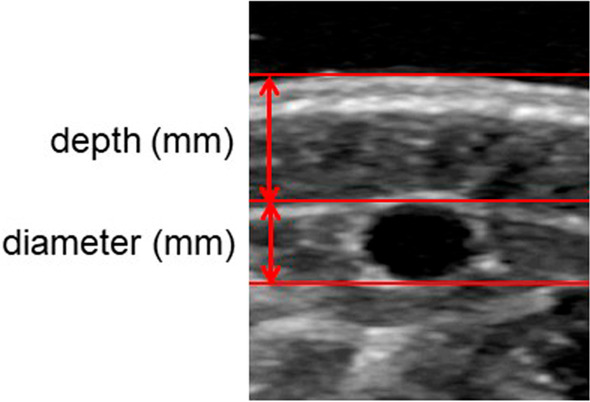
Ultrasound image of the superficial vein. Venous depth is the distance from the skin surface to the intima, and venous diameter is the distance between the intima

Figure 1 shows the analysis of the vein depth and diameter. First, a line was drawn on the skin surface, and the intima of the vein was traced manually while moving the line downward by 1 pixel. The vein depth was defined as distance from the skin surface to the intima and the vein diameter as the distance between the intima. The acquired images were analyzed blindly and randomly, and the participants were de-identified.

### Statistical analysis

The intraclass correlation coefficient (ICC) was calculated for short-term and long-term reproducibility of measurements. Strictly, the ICC shown in this study is the reproducibility of one measurement (ICC [1,1]) and does not include interrater variation. The coefficients of variation (CVs) for the intra-individual variation in the depth and diameter measurements were also analyzed according to a previous study [29]. For the results of both vein depth and diameter, the average of five measurements for each experimental day was determined, and Bland–Altman plots were performed on the difference between 2-day measurements. A fixed bias and a proportional bias were tested by a paired t-test and Pearson correlation coefficient (r). Pearson’s correlation coefficients were also calculated to examine the relationship between vein depth and intra-individual variation of the diameter. Furthermore, the correlation of the vein depth and diameter against the body composition were also analyzed. Statistical analyses were performed using R software, version 4.1.0.

## Results

Tables 2 and 3 show the results for depth and diameter, respectively. The mean vein depths ranged from 2.43 to 2.59 mm. Short-term and long-term ICCs ranged from 0.94 to 0.96 and 0.88, respectively. By contrast, vein diameters averaged 2.48 at 2.53 mm. Despite the high short-term ICC of 0.94–0.97, it decreased between days, resulting in a long-term ICC of 0.67. Tables 2 and 3 show the CVs for the depth and diameter measurements, respectively. Vein depth exhibited a 6–7% intra-individual variation for both long- and short-term variabilities. The CVs for the vein diameter were approximately 5–6% and 9% for the short-term and long-term variabilities, respectively.

**Table 2:** Vein depth measurements and their reproducibility

	Mean ± SD (mm)	ICC	95% CI	Intra-individual CV (%)
Shor term (day 1)	2.43 ± 0.90	0.96	0.93–0.98	6.56
Short term (day 2)	2.59 ± 1.05	0.94	0.89–0.97	6.27
Long term (days 1–2)	2.51 ± 0.98	0.88	0.75–0.95	6.34

*SD* standard deviation; *ICC* intraclass correlation coefficient; *CI* confidence interval; *CV* coefficient of variation

**Table 3:** Vein diameter measurements and their reproducibility

	Mean ± SD (mm)	ICC	95% CI	Intra-individual CV (%)
Short term (day 1)	2.48 ± 0.67	0.94	0.90–0.97	6.07
Short term (day 2)	2.53 ± 0.73	0.97	0.94–0.98	4.82
Long term (days 1–2)	2.51 ± 0.70	0.67	0.38–0.85	8.88

*SD* standard deviation; *ICC* intraclass correlation coefficient; *CV* coefficient of variation

**Fig. 2:**
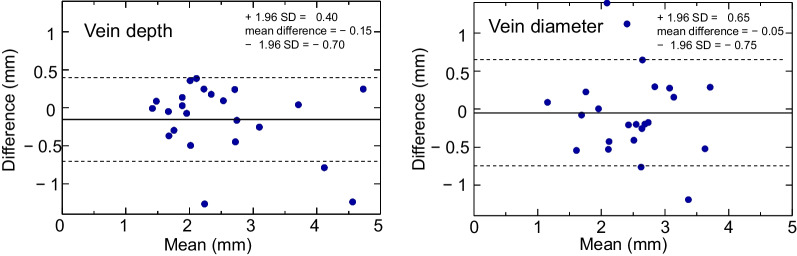
Bland–Altman plots for vein depth (left) and vein diameter (right). The differences (y axis) between the mean values obtained in 2 experimental days were plotted against their mean value (x axis). Dashed lines designated upper and lower limits of 95% confidence interval of the difference. Fixed and proportional biases were not found in either vein depth or vein diameter

The Bland–Altman plots for vein depth and diameter are demonstrated in Fig. 2. For vein depth, there was no significant difference between two experimental days (p = 0.12), thus no fixed bias in vein depth. As for proportional bias, a positive correlation (r = − 0.30) was found although it is not significant (p = 0.16). As for vein diameter, fixed bias (p = 0.69) and proportional bias (r = − 0.13, p = 0.54) were also insignificant.

**Fig. 3:**
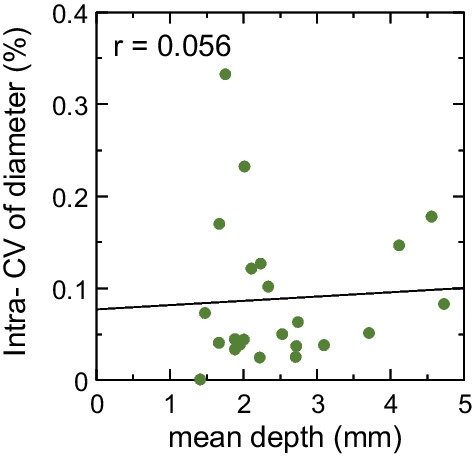
Correlation between the mean vein depth and mean intra-individual coefficient of variation (intra-CV) of the vein diameter. No correlation was found between the mean vein depth and mean intra-CV of the vein diameter (r = 0.056)

A correlation between the mean vein depth and intra-individual CV of the vein diameter is shown in Fig. 3. The correlation coefficient (r) was 0.056; thus, the correlation was not significant.

**Fig. 4:**
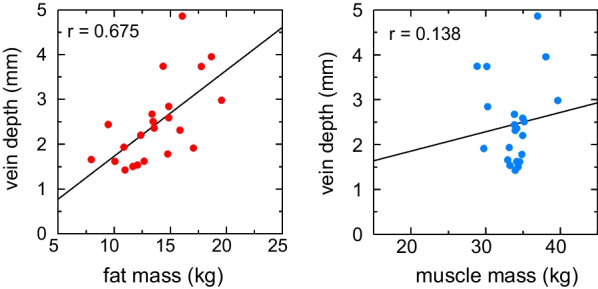
Correlation between fat mass, muscle mass, and vein depth. Vein depth positively correlated with fat mass (r = 0.638) but not with muscle mass (r = 0.138)

**Fig. 5:**
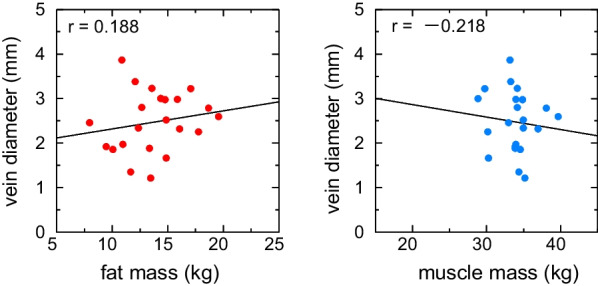
Correlation between fat mass, muscle mass, and vein diameter. The diameter did not correlate with fat or muscle mass (r = 0.188 and − 0.218, respectively)

Body fat mass and total body muscle mass were examined in relation to the vein depth and diameter, respectively (Figs. 4 and 5). Body fat mass was significantly positively correlated with the vein depth (r = 0.675, 95% CI = 0.281–0.830) but not with the muscle mass (r = 0.138, 95% CI = − 0.318–0.515). Furthermore, neither body fat mass nor muscle mass correlated with the vein diameter.

## Discussion

In this study, to examine the reproducibility of the ultrasound measurement of the vein diameter and vein depth, repeated measurements were performed with short- (10 s) and long-term (24 h) intervals. For short-term reproducibility, ICCs ranged from 0.94 to 0.97 for vein diameter and 0.94–0.96 for vein depth. The long-term reproducibility was 0.67 for vein diameter and 0.88 for depth. Short-term reproducibility did not differ much between the vein diameter and depth, but the long-term reproducibility of vein diameter was considerably lower than that of depth.

This study also examined the relationships between body composition, vein depth, and vein diameter. Vein depth and body fat mass positively correlated, whereas vein diameter did not correlate with either body fat mass or total body muscle mass.

Sharp et al. [30] reported that the body mass index (BMI) had little effect on the vein diameter. Kim et al. [31] did not find a relationship between the vein diameter and BMI, although BMI influenced depth. The results of the present study were consistent with their findings.

Since fat mass is unlikely to change in a few days, it appears reasonable that depth is more reproducible over time than diameter.

As shown in Table 3, the peripheral forearm vein diameter indicated approximately 5% (short-term) or 9% (long-term) variability. Previous studies have demonstrated 18–75% variation in the diameter of the inferior vena cava and 7–10% for the veins of the lower extremities [22, 23]. Although the methods of measurement and analysis differed between the previous study and the present study, the variation of the forearm superficial vein was much smaller than that of the inferior vena cava and almost the same as that of lower extremity veins when roughly recognized. Venous variation might be greater in the center and smaller in the periphery. Most studies on venous variability have focused on the central vein, such as the inferior vena cava [22, 32]. In contrast, the present study focus was on the peripheral superficial veins. The novelty of the current study is the quantitative demonstration of the variation of the forearm superficial veins.

The main cause of the variation in the diameter of the vein (e.g., inferior vena cava, axillary vein**)** is respiration [22, 32]—the diameter of the vein is known to contract with inspiration and to dilate with expiration [33, 34]. Therefore, respiratory modulation would be expected in the peripheral superficial vein as same as the inferior vena cava. We cannot determine whether the variability could be attributed to respiratory modulation because respiration was not measured in this study. Further studies on the effect of respiration on peripheral superficial vein diameters are required.

This study has some limitations. First, the presented results on short-term reproducibility are limited to variations of a few minutes. In this study, long-term measurements were conducted at the same time on different days; therefore, the results excluded the effects of diurnal variation. Further research is needed to determine the factors that affect the reproducibility of vein diameter measurements. Second, the participants of this study were limited to young healthy women. Sharp et al. [30] reported that men tend to have larger venous diameters than women. Therefore, there could be sex differences also in diameter variability. Future studies are expected to take into account the influence of demographics such as age and race in addition to sex.

## Conclusions

Short-term measurements of the depth and diameter of peripheral superficial veins showed excellent reproducibility. The vein diameter indicated a slightly lower long-term reproducibility than the short term. The larger day-to-day variations in vein diameter can be attributed to the variation in the physiological condition of the participants rather than to the instability of the measurement. Therefore, ultrasound measurement of the peripheral superficial vein is sufficiently reliable.

## Data Availability

The datasets used and/or analyzed in the current study are available from the corresponding author on reasonable request.

## Abbreviations

ICC: Intraclass correlation coefficient
CV: Coefficient of variation
SD: Standard deviation
CI: Confidence interval
BMI: Body mass index
